# Application of Machine Learning Techniques for Characterization of Ischemic Stroke with MRI Images: A Review

**DOI:** 10.3390/diagnostics12102535

**Published:** 2022-10-19

**Authors:** Asit Subudhi, Pratyusa Dash, Manoranjan Mohapatra, Ru-San Tan, U. Rajendra Acharya, Sukanta Sabut

**Affiliations:** 1 Department of Electronics & Communication Engineering, ITER, SOA Deemed to be University, Odisha 700107, India; 2 Department of Computer Science and Engineering, Heritage Institute of Technology, Kolkata 700107, India; 3 Department of Radiology, KIMS, KIIT Deemed to be University, Odisha 751024, India; 4 Department of Cardiology, National Heart Centre Singapore, Singapore 169609, Singapore; 5 Cardiology, Duke-NUS Medical School, Singapore 169857, Singapore; 6 Department of Electrical and Computer Engineering, Ngee Ann Polytechnic, Singapore 599489, Singapore; 7 Biomedical Engineering, School of Social Science and Technology, Singapore University of Social Sciences, Singapore 599494, Singapore; 8 International Research Organization for Advanced Science and Technology (IROAST), Kumamoto University, Kumamoto 860-8555, Japan; 9 Department Bioinformatics and Medical Engineering, Asia University, Taizhong 41354, Taiwan; 10 School of Management and Enterprise, University of Southern Queensland, Toowoomba, QLD 4350, Australia; 11 School of Electronics Engineering, KIIT Deemed to be University, Odisha 751024, India

**Keywords:** brain stroke, medical imaging, computer-aided-diagnosis, machine learning, decision support system, artificial intelligence

## Abstract

Magnetic resonance imaging (MRI) is a standard tool for the diagnosis of stroke, but its manual interpretation by experts is arduous and time-consuming. Thus, there is a need for computer-aided-diagnosis (CAD) models for the automatic segmentation and classification of stroke on brain MRI. The heterogeneity of stroke pathogenesis, morphology, image acquisition modalities, sequences, and intralesional tissue signal intensity, as well as lesion-to-normal tissue contrast, pose significant challenges to the development of such systems. Machine learning (ML) is increasingly being used in predictive neuroimaging diagnosis and prognostication. This paper reviews image processing and machine learning techniques that have been applied to detect ischemic stroke on brain MRI, including details on image acquisition, pre-processing, techniques to segment, extraction of features, and classification into stroke types. The main objective of this work is to find the state-of-art machine learning techniques used to predict the ischemic stroke and their application in clinical set-up. The article selection is performed according to PRISMA guideline. The state-of-the-art on automated MRI stroke diagnosis, with a focus on machine learning, is discussed, along with its advantages and limitations. We found that the various machine learning models discussed in this article are able to detect the infarcts with an acceptable accuracy of 70–90%. However, no one has highlighted the time complexity to predict the stroke in the model developed, which is an important factor. The work concludes with proposals for future recommendations for building efficient and robust deep learning (DL) models for quantitative brain MRI analysis. In recent work, with the application of DL approaches, using large datasets to train the models has improved the detection accuracy and reduced computational complexity. We suggest that the design of a decision support system based on artificial intelligence (AI) and clinical data presenting symptoms is essential to support clinicians to accelerate diagnosis and timeous therapy in the emergency management of stroke.

## 1. Introduction

Stroke is a leading cause of death and disability among adult survivors worldwide [1]. A stroke can be either ischemic or hemorrhagic in nature. Stroke has heavy physical, social, economic, and emotional burdens on patients and their families [2]. According to the WHO, globally, each year, approximately 15 million people suffer brain stroke, of which one-third die and the remaining become permanently disabled [1,3]. In India, the prevalence rates of stroke in rural and urban areas range from 55 to 388 per 100,000 and 45 to 487 per 100,000 [4,5], respectively. The majority of stroke is attributable to high blood pressure [6]. Other contributory risk factors include an unhealthy lifestyle and smoking. Stroke incidence and mortality can be prevented by effectively controlling risk factors, such as hypertension, hyperlipidemia, and tobacco consumption. As a result, stroke incidence has decreased by approximately 10% between 1990 and 2010 in developed countries. The preventive impact is, unfortunately, less felt in developing countries, where the incidence has increased by 10% in the same period [3].

### 1.1. Classification of Stroke

According to pathogenesis, strokes can be classified into hemorrhagic and ischemic types [7]. Approximately 70% of all stroke cases are ischemic in nature and the condition is observed with neurological deficit which persists beyond 24 h or is interrupted by death within 24 h [8]. Approximately 12% of all strokes are hemorrhagic, which comprise 9% intracerebral and 3% subarachnoid hemorrhages. Hemorrhagic strokes are caused by the rupture of cerebral blood vessels or vascular malformations which bleed into adjacent brain tissues, affecting their function and more likely to lead to death than permanent disability. In contrast, ischemic strokes occur due to blockage in blood vessels that supply the brain, and by far represent the majority type. Ischemic strokes can be classified according to their clinical manifestation. In the Oxfordshire Community Stroke Project [9], stroke episodes were classified based on initial symptoms and their severity into four groups that are predictive of stroke extent and affected brain region, underlying cause, as well as prognosis: total anterior circulation stroke syndrome (TACS); partial anterior circulation stroke syndrome (PACS); lacunar stroke syndrome (LACS); and posterior circulation stroke syndrome (POCS). LACS, the commonest type, occurs due to blockages in small arteries that supply deep structures of the brain. Patients characteristically suffer pure motor or sensory deficit, sensorimotor deficit, or ataxic hemiparesis. TACS occur when blood supply to the anterior and middle cerebral arteries on either side of the brain becomes compromised, which results in unilateral paralysis. PACS is a less severe form of TACS, in which some but not all symptoms associated with TACS are manifest. POCS is caused by the reduced blood supply to the posterior cerebral artery on one side of the brain. Clinically, the survivors suffer neurological deficit with abnormal body function.

### 1.2. Acute Stroke Imaging

**Figure 1:**
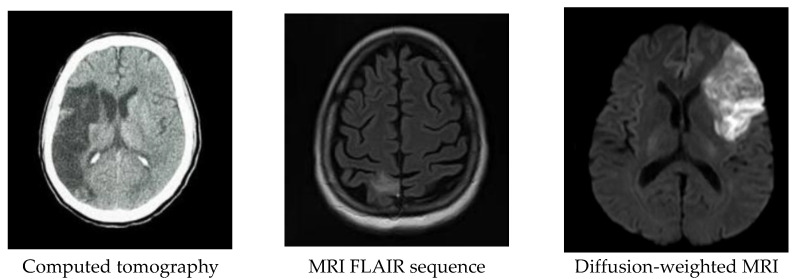
Neuroimaging in acute ischemic stroke of different patients. On computed tomography, infarct is seen as a hypointense region (**left**). With MRI, signal contrasts between different tissues can be amplified using different pulse sequences. With fluid attenuated inversion recovery (FLAIR), the infarct is depicted as a bright signal against surrounding brain tissue as well as the dark signal-suppressed cerebrospinal fluid (**center**). With DWI, the infarct tissue exhibits less signal decay as water diffusion becomes restricted, which shows up as a hyperintense area (**right**).

In stroke, the functional deficit corresponds to the site and extent of the ischemic or hemorrhagic brain lesion. The early detection of stroke and its type is important to clinicians for deciding an optimal management process. Computed tomography (CT) and MR imaging are standard investigation tools for excluding brain hemorrhage as well as for characterizing ischemic lesions and quantifying potentially salvageable tissue at risk [10]. CT is exquisitely sensitive to the presence of hemorrhage, whereas MRI is the most sensitive technique for the early identification of ischemic stroke. MRI, which exploits different pulse sequences to enhance the signal contrast between normal and infarct tissues, is the most sensitive technique for early stroke identification [11,12]. The diffusion-weighted imaging (DWI) sequence of MRI is commonly used to identify the area of hypoperfusion, i.e., area at risk, and the irreversibly damaged infarct core, respectively [13]. In the DWI sequence, the intensity of signal exponentially decays with the rate of diffusion in a voxel [14]. Acute brain ischemia induces temporal changes in the intracellular sodium content of the injured brain tissue, and intracellular water movement becomes restricted consequently. DWI is extremely sensitive to perturbed water diffusion, manifesting as a bright signal perceptible within minutes of acute ischemic stroke [15,16,17,18]. Mismatch between areas of hypoperfusion and DWI-assessed acute infarct-manifest as a penumbra surrounding the infarct core, respectively, signifying potential salvageability [19]. The contrast of the same tissue can be varied by varying the b-value in sequences [20,21]. Increase in b-value attenuates the intensity of the signal which helps to improve the contrast of the lesion [22,23]. Figure 1 shows examples of CT and MRI images in acute ischemic stroke collected from different patients.

### 1.3. Image Segmentation 

Image segmentation aims to represent an image in a more meaningful way for analysis. It involves the partitioning of the image either manually or automatically into various regions that share similarities in signal intensity and properties and can be used for localizing lesions in the brain [24]. Automated image segmentation is an important step in the processing of brain images which facilitates lesion detection and quantification of the extent. This information is obligatory for accurate disease prognostication and optimal clinical management.

### 1.4. Computer Aided Diagnosis (CAD) for Detection of Stroke

**Figure 2:**
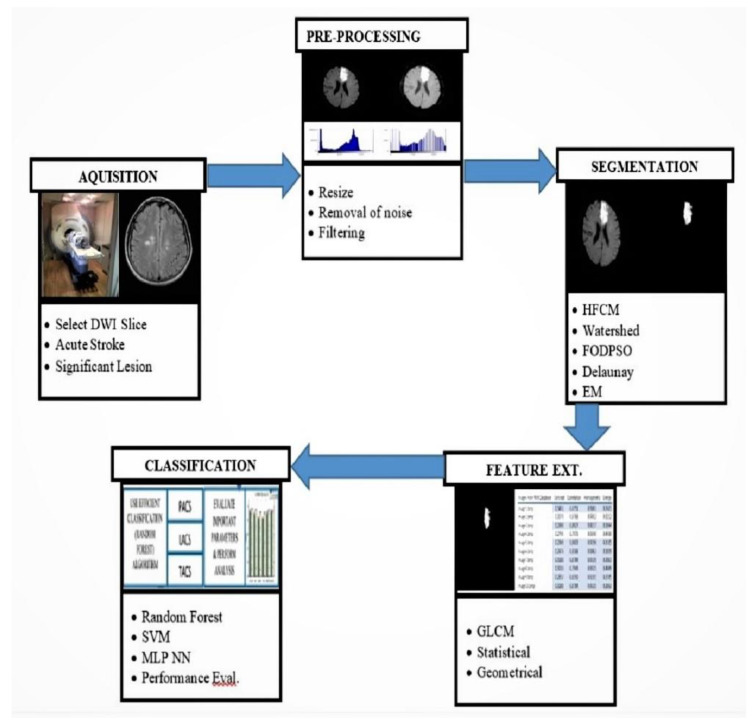
Flowchart of typical computed-aided diagnosis system for end-to-end stroke detection. DNN, deep neural network; NN, neural network.

The CAD process has been applied in medical imaging for disease detection, prognostication, decision support for guiding treatment, and therapeutic monitoring [25]. In MRI, manual segmentation exacts high time costs as experts have to scrutinize multiple images of the brain acquired in various orientations using different pulse sequences. Moreover, there is potential for inter-and intra-observer biases [26,27,28]. Semi-automated and automated machine learning-based CAD systems for identifying and segmenting ischemic stroke lesions can surmount these limitations, facilitating high throughput screening of images for faster, reproducible, and more sensitive detection of ischemic stroke lesions [29]. Automated delineation of the exact topology of stroke lesions facilitates quantitative analyses of infarct size and/or salvageability, which are useful for prognostication and therapeutic decision-making. A typical CAD system for stroke comprises distinct sequential stages (Figure 2):

- Image acquisition and pre-processing stage: For acute ischemic stroke detection, DWI sequence of MRI is the modality of choice. In the pre-processing stage, the images were first normalized using linear scaling, followed by background removal using simple thresholding. The quality of the image is enhanced further with contrast-limited adaptive histogram equalization (CLAHE).
- Image segmentation: Lesions are segmented using various methods, including clustering, watershed, and optimization and classified with different classifiers.
- Features extraction: Extracted statistical or morphological features are used as input to the classifiers for classification of stroke and its sub-types.
- Classification: Rule-based classifiers, such as neural network, support vector machine (SVM), decision tree, and random forest classifier, are implemented to classify ischemic brain lesions according to established standards, e.g., the Oxfordshire Community Stroke Project classification scheme.

## 2. Materials and Methods

### 2.1. Article Search Strategy

We performed a systematic review of articles according to the PRISMA guidelines [30]. An extensive search strategy was developed for this study consisting of different combinations of the following keywords: “brain stroke”, “ischemic stroke”, “haemorrhage stroke”, “magnetic resonance imaging”, “detection”, “segmentation”, “lesion”, “infarct identification”, “segmentation of lesion”, “prediction of ischemic tissue”, and “machine learning for stroke classification”. The database search was conducted systematically only for published articles from 1990 to till May 2022 using search engines such as IEEE Xplore, Wiley, Science Direct and Springer. PubMed, Embase, Web of Science (ISI), and the Cochrane Library were also used separately for search. Additional articles were collected by reviewing the reference sections of the screened articles. All articles that reported the brain stroke patients were included during the initial search.

### 2.2. Article Selection

**Figure 3:**
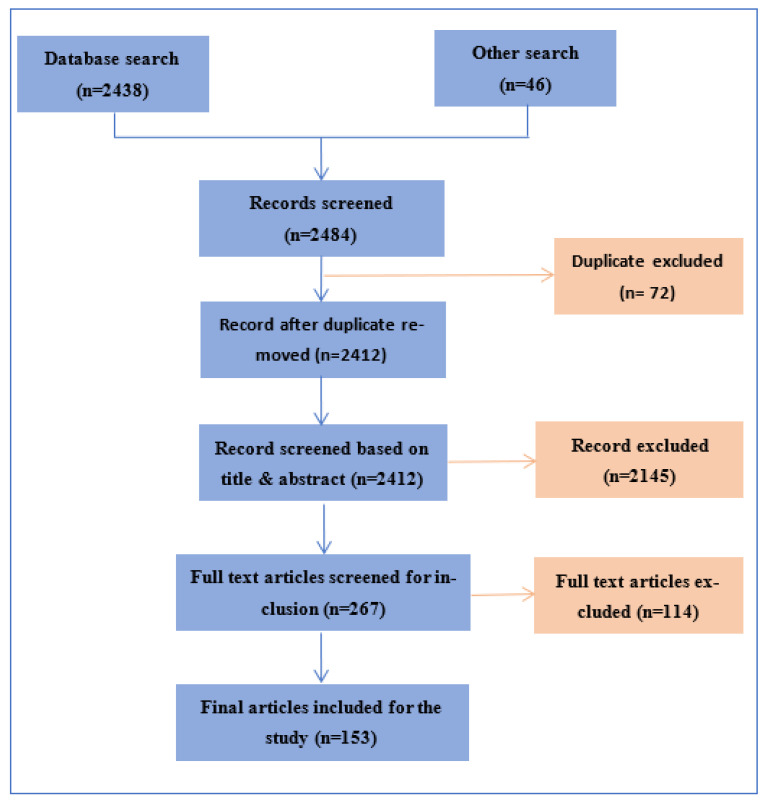
Flow chart for article selection process.

The articles published in English between 1990 and April 2021 which are related to the subject area of review and a few earlier articles describing the concepts of the methods were considered. The electronic search strategy yielded 2484 studies, and we excluded 2145 studies after screening the title and abstract of the paper which did not meet criteria, and specifically where the segmentation approach was not described. After reviewing full texts, another 114 articles were excluded and, finally, 153 suitable studies were included in the systematic review process based on relevance, methods, and technical details of implementation. The article selection process is shown in Figure 3. We collected all publications covering this subject related to the segmentation and classification of brain stroke using MRIs. We excluded case reports and all articles that included animal studies. Authors verified the title and abstract of each article. Only relevant articles were then considered for full-text screening for inclusion in the study.

## 3. Results

We identified several research articles that applied machine learning techniques for ischemic stroke detection. The methods described, broadly segregated into segmentation techniques and machine learning approaches, are reviewed in the following sections.

### 3.1. Segmentation Techniques

#### 3.1.1. Overview

Manual segmentation of infarcts in MRI data is a difficult, time-consuming, and challenging task [31,32]. Many studies have reported automated methods for object recognition and classification [33,34]. Specifically, methods proposed for image segmentation include edge-based, region-based, thresholding, clustering-based, and supervised methods [35,36,37,38,39,40]. Semiautomatic methods are applied for segmentation in medical image analysis. In [41], region-growing based on image signal intensity was used to extract a connected region by manually seeding a point within the region of interest [41]. In [42], a rule-based expert system is used for automatic classified stroke lesions on MRI data using seeded region-growing method. Unsupervised learning methods have also been used successfully to segment ischemic infarcts [43,44,45]. James et al. [46] used a histogram partitioning-based approach to segment the infarct core and the penumbra in DWI sequences. Mangla et al. [47] used various techniques to characterize cortical and subcortical border zones of infarct on MRI according to the underlying pathophysiologic processes. In [48], the background voxels and brain tissue were separated on MRIs by thresholding and classification of tissue using fuzzy C-means clustering. Martel et al. [49] measured infarct volume in MRI using an adaptive thresholding algorithm and Markov random fields. Usinskas et al. [50] presented an unsupervised method to segment ischemic stroke regions based on computing mean and standard deviation features. Several automated methods have been published for segmenting infarcts in MRI images [51,52,53,54,55]. Li et al. [56] reported an unsupervised method based on multistage processes that included tensor field calculation, diffusion anisotropy measurement, adaptive multiscale statistical classification for segmentation of infarct volume, and partial volume voxel re-classification. Prakash et al. [57] segmented the infarct on MRIs using a probabilistic neural network and adaptive Gaussian mixture model. Hevia-Montiel et al. [58] used a nonparametric density estimation method to segment infarct on DWI sequences. Gupta et al. [59] use DWI images to detect infarct based on its intensity characteristics. Shen et al. [60] reported a method to detect infarct based on separation of the voxel intensity and spatial tissue distribution. 

#### 3.1.2. Clustering

**Figure 4:**
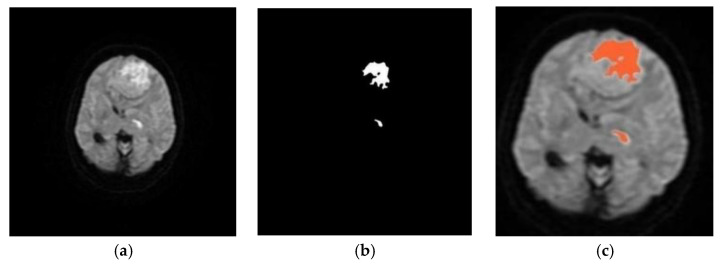
Original DWI image (**a**), and morphological binary image (**b**), detected infarct marked as red (**c**) [78].

The fuzzy C-means clustering (FCM) approaches have been successfully applied for medical image analysis as such approaches retain valuable information from the original image [61,62,63]. However, standard FCM can fail to produce accurate results when there is excessive image intensity inhomogeneity or noise [64,65,66]. Thus, the modified FCM is used to perform segmentation on noisy images [67,68]. Griffis et al. [69] combined naive Bayes classification and cluster-extent thresholding for the automated detection of stroke lesions on T1-weighted images with Dice similarity and Pearson’s coefficients of 0.66 and 0.97, respectively. Seghier et al. [70] segmented the chronic lesions on T1-weighted MRI using fuzzy clustering. The FCM algorithm can also be improved by partitioning the images in a meaningful region [71,72]. He et al. [73] incorporated constraints into the FCM algorithm to perform brain tissue segmentation on diffusion tensor MRI. In the adaptive FCM algorithm, the objective function gradually becomes better for improving segmentation [74,75]. In [76,77], fuzzy local information C-means (FLICM) improved the performance in terms of noise and computational time. In [78], a hybrid approach was presented combining the K-means and FCM algorithm, which improved the accuracy in detecting brain infarct with less computational costs (Figure 4).

#### 3.1.3. Watershed Transformation (WT)

**Figure 5:**
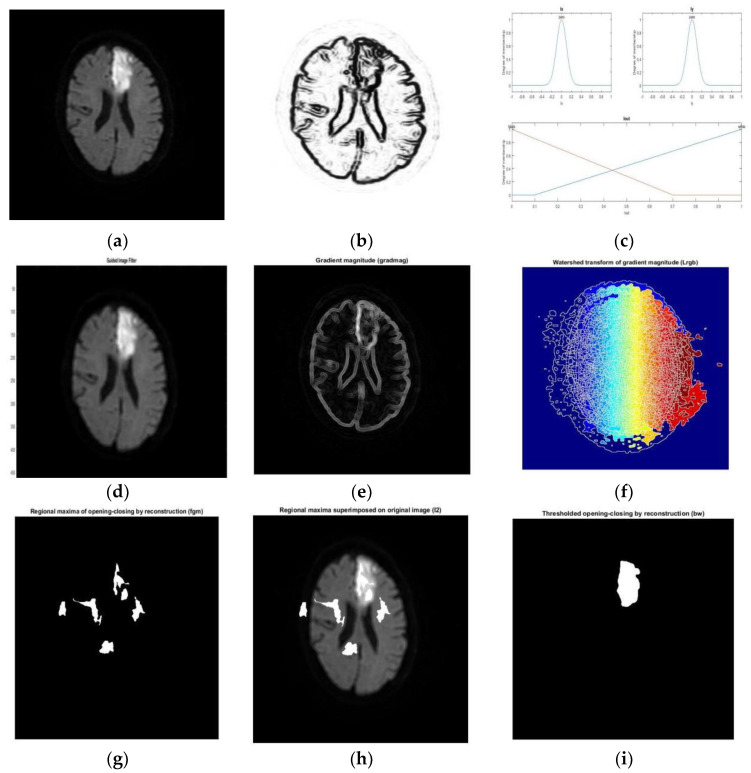
Watershed segmentation of brain infarcts: (**a**) original image (**b**) Edge detection with fuzziness; (**c**) Membership function; (**d**) Output of guided filter; (**e**) Gradient magnitude image; (**f**) output of watershed transform; (**g**) Reconstruction of image; (**h**) Superimposed image on original image; (**i**) Final detected infarct with morphological operation [89].

The WT algorithms are widely used in image segmentation and produce a sharp boundary of the object in low-contrast images [79,80,81]. The drawbacks of WT, e.g., over-segmentation, can be eliminated by using appropriate filters [82,83]. In [84], a difficult region comprising gray and white matter of the brain was segmented with a directional WT algorithm on noisy 3D brain MRI images [85,86]. In [87], the classification accuracy of 0.90 was achieved with the morphological operation of a WT model. An interactive multiscale WT algorithm could accurately segment brain tumors on MRI compared to manual segmentation [88]. A segmentation approach that combines WT with random forest algorithm provides better detection of infarcts with accuracy of 95% in DWI of the brain [89] (Figure 5).

#### 3.1.4. Intelligent Optimization

**Figure 6:**
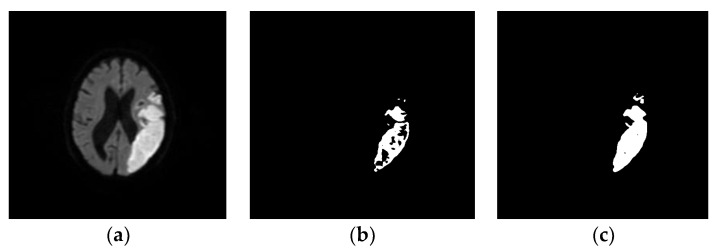
Large ischemic stroke lesion is seen on the original DWI (**a**), with lesion detected using PSO (**b**) and fractional-order DPSO (**c**) [99].

Optimization techniques, such as expectation-maximization (EM) and optimization via graph cuts, improved segmentation accuracy on MRI. An optimization approach achieved a similarity index of 0.849 in segmenting infarct volumes with the fast computational time of approximately 3–4 min [90,91]. In [92], an entropy-based maximization method with a set threshold value and particle swarm optimization (PSO) was able to separate lesions from healthy tissue on brain MRI. A novel automated intensity-based method based on the histogram-gravitational optimization algorithm (HGOA) attained 0.91 for segmenting stroke lesions on single-modality T1-weighted brain MRI [93]. In [94], a fully automated discrete curvelet transformation-based approach was effective for detecting ischemic stroke lesions on brain MRI. Pham et al. [95] integrated fuzzy entropy clustering into an improved PSO model for segmenting brain MRI. Ghosh et al. [96] used adaptive thresholding to segment ischemic lesions on T2-weighted MRI of animal models. Biology-inspired algorithms have also been used for image segmentation [97,98]. Subudhi et al. [99] proposed a novel method based on Darwinian particle swarm optimization (DPSO) that could identify stroke lesions on brain DWI with 90.23% accuracy using SVM classifier. Couceiro et al. [100] presented fractional-order DPSO (FODPSO), an extension of DPSO, which could control the convergence rate successfully (Figure 6).

**Figure 7:**
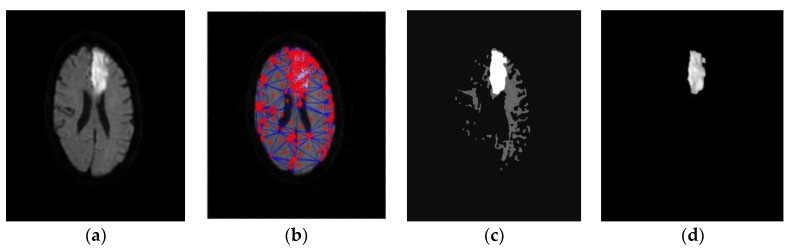
Ischemic stroke lesion is seen on the original DWI (**a**), Voronoi cells are generated (**b**), and Delaunay triangulation applied (**c**), The extracted lesion detected using Delaunay triangulation FODPSO (**d**) [114].

Expectation-maximization (EM) algorithms were also used for image segmentation with different probabilistic models [101]. In the case of multiple local maxima, they may not converge to the global maximum [102]. Yoon et al. [103] detected and classified the lesions with adaptive FCM on a large axial brain MRI dataset. Niu et al. [104] used a random swap EM algorithm for color image segmentation. Huang and Liu used EM to estimate the Gaussian parameters for classifying color image [105]. Mahjoub and Kalti [106] segmented images using a Bayesian algorithm-based finite mixture model, in which an EM algorithm was used estimate parameters of Gaussian mixture model. Marroquin et al. [107] applied an EM algorithm for efficient automated segmentation of the brain from non-brain tissue on 3D MRI data. Tian et al. [108] developed a hybrid genetic algorithm-variational EM (GA-VEM) model that improved the performances of segmentation in brain in MRI images. In [109], the noise effects were reduced with spatial information and bias correction of EM and FCM algorithms thereby improved the accuracy in segmentation of gray and white matter on brain MRI [109]. Kwon et al. [110] segmented the brain lesions by combining WT and EM algorithms with a clustering approach. Rouainia et al. [111] built a statistical model from the data, and successfully applied the EM algorithms to detect brain lesions on MRI. It is necessary to have sharp segmentation of lesion boundary to understanding the stroke deficit in brain image [112,113] until recently. Using a novel methodology, Subudhi et al. [114] developed a Delaunay triangulation and optimization-based system that detected the brain infarct with a better accuracy of 95% (Figure 7).

### 3.2. Machine Learning

Table 1 summarizes studies that used machine learning methods to detect acute ischemic stroke lesions. The method was evaluated for both the segmentation and classification of stroke lesions using measured parameters, e.g., sensitivity, accuracy, and Dice index.

Intelligent classifiers, such as artificial neural networks (ANN), SVM, and decision tree methods have been used successfully for brain stroke detection and classification [128,129,130]. Abedi et al. [128] developed an ANN model to recognize acute cerebral ischemia. Kasasbeh et al. [131] detected infarcts in acute stroke patients in DWI sequences with better accuracy. Wilke et al. [115] used semi-automated and automated approaches for detected chronic stroke lesions on MRI using fuzzy clustering. Mitra et al. [116] used an automated method based on the Bayesian–Markov random field for classifying chronic infarcts on FLAIR MRI. Chyzhyk et al. [132] reported an effective segmentation approach of infarcts using active learning of classifiers on multimodal MRI data.

Various techniques based on machine learning approaches are used to determine the time since stroke onset based on imaging features and decision support tool for planning of stroke treatment [133,134]. Deep learning segmented the acute infarcts accurately on DWI sequences of MR images with a Dice coefficient of 0.79 [135]. Bhattacharya et al. [136] used an antlion optimization algorithm with deep neural network (DNN) to select optimal hyperparameters that improved the quality of stroke data and classification. Maier et al. [137] tested their ischemic stroke segmentation model, which combined linear models, random decision forests, and CNNs, on 37 MRI datasets. They concluded that high-level machine learning methods like random forest and CNN can classify more accurately than standard classification methods. Guibas and Stolfi [138] introduced clustering scheme on brain tissues having similar characteristics with a 3D Delaunay triangulation approach. 3D geometrical modeling of human tissues. A 3D Delaunay triangulation is used to segment non-overlapping regions having similar characteristics in CT/MR images [113,139]. Pennisi et al. [112] used the DT for detecting skin cancer lesions, based on geometrical and color features. Recently ML approaches using DNNs have been applied for image segmentation, automated feature extraction in brain images [140]. A deep extreme learning method was applied effectively for the classification of pathological brain lesions on multiclass MRIs [141]. In [120], a general method was applied to segment hyperacute ischemic infarct by extracting multiple features and classified using a random forest classifier with a Dice coefficient of 0.774. Mah et al. [121] proposed a high dimensional algorithm that to quantify the ischemic damage on MRI with sensitivity and similarity index of 0.93 and 0.73, respectively. In [122], the template-based FCM method achieved a Dice index of 0.687 in segmenting brain lesions in MRI images. In [123], the learning algorithms along with SVM classifiers detected the infarcts with a Dice coefficient of 0.73 on T1-weighted images. 

**Figure 8:**
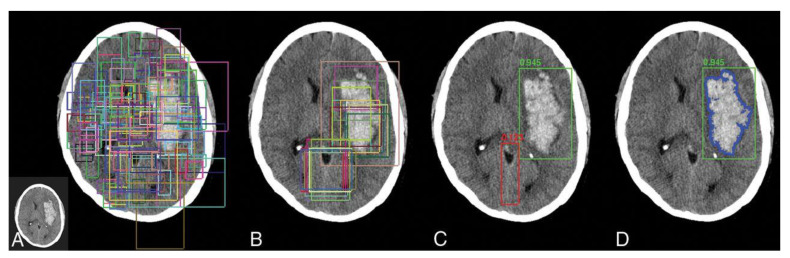
Overview of the mask R-CNN approach. (**A**), The bounding boxes indicates the presence of abnormality; (**B**), Generation of region; (**C**), Composite region proposals; and (**D**), Segmentation masks for hemorrhage region. Source [150].

Artificial intelligence (AI) is increasingly being used to automated stroke diagnosis on brain imaging. In the coming years, AI and smart technology can be integrated into stroke care by neurologists. It will improve the diagnosis and treatment process there by reducing morbidity and mortality [142,143]. Deep learning has been applied for image segmentation, automated featurization, and multimodal prognostication in stroke management [144]. Haskin et al. [145] reviewed the use of deep learning approaches for medical image analysis. Kaur et al. [146] explored different deep and transfer learning models for classification of pathological brain images. AlexNet with transfer learning yielded the best results with accuracy of 100% with less computational costs compared other models. Winzeck et al. [147] used an ensemble of convolutional neural networks to train the model that combinations 116 different images of DWI, ADC, and low b value-weighted sequences. Model produced better segmentation accuracy on acute infarcts. Xue et al. [148] proposed a CNN model with multi-modal path for automating segmentation of stroke lesion. The model was designed with nine series UNets with the input of 2D slices, and examined with a final lesion mask of 3D CNN model. Liu et al. [127] developed a deep learning tool and the trained the model on 2348 images of DWI sequences collected from acute and sub-acute patients having ischemic strokes. The DAGMNet model resulted better performance than UNet, with higher Dice index scores of 0.74 and higher precision of 0.76. Bridge et al. [149] used a deep learning model trained on 6657 DWI sequences could segment the infarcts with Dice coefficient 0.776 [149]. Chang et al. [150] designed a customized deep learning approach, a hybrid 3D/2D based CNN network for hemorrhagic evaluation in CT images, and quantified the hemorrhagic lesions on NCCT images with Dice score of high accuracy 0.93. The R-CNN mask provides an efficient model for object classification and segmentation and the results are depicted (Figure 8). It is concluded that the brain infarct can be detected accurately on MRI images using a machine learning model in real-world clinical scenarios.

## 4. Discussion

It is a challenging task to analyze the infarcts using medical imaging which comprises various steps from image acquisition to the classification of stroke types. This makes the aforementioned steps more complicated. So far, there are no particular tools available to confirm the types of stroke, severity of infarcts, and chances of recovery. The clinicians rely on the image analysis performed manually, which is a challenging issue and leads to inter-reader variability. Therefore, computer assisted analysis is a growing area of research in brain imaging. Table 1 summarizes the main characteristics of the review articles discussed in the previous sub-sections. It includes important articles that describes guidelines and objectives of the study and important contribution to detect the infarcts in brain imaging. A fast and accurate automated CAD system for brain MRI analysis will facilitate the timely management of stroke. In this review, we have surveyed large number of research papers to find different imaging techniques that have been applied successfully to detect the brain stroke on MRI images. Various researchers presented automatic methods to detect and classify infarcts using MRI (T1, T2, and FLAIR images) as it is radiation-free and it provides good contrast. Such approaches mainly focused on increasing classification accuracy by extracting different features from the readouts of various brain MRI sequences. To optimize the lesion detection, some reports also explored of image fusion across modalities like CT, MRI, and functional MRI [130,146]. Of note, the quantification of infarct volume is important for prognostication. The volume can be estimated by forming a 3-D structure reconstructed from segmented lesions across contiguous MRI slices [84,107].

Conventional methods may fail to capture small foci of infarct on brain MRI compared with expert delineation for stroke segmentation [115]. This has motivated researchers to explore machine learning models, which have recently gained prominence in medical diagnostic decision-making. The complex data structure in neuroimaging is a significant challenge that machine learning approaches to stroke classification have to overcome. In general, the performance of machine learning models for stroke lesion segmentation and classification has been salutary (Table 1), which supports their application for the further development of decision support tools in stroke diagnosis and treatment selection. In an automatic approach, Maier et al. [117] classified the subacute lesions of ischemic stroke using intensity-based features and extra trees framework with a Dice coefficient of 0.65 tested on 37 DWI sequences. They also obtained acceptable classification of lesions with SVM [118], but it was time-consuming. Griffs et al. [119] used a naive Bayes classifier to detect infarcts on T1-weighted and obtained good segmentation accuracy with Dice index of 0.66. Of note, the performance evaluation of CAD models had been mostly carried out for sensitivity and accuracy measures, whereas the computational complexity of the various methods was largely under-reported. As timely diagnosis is paramount, real-time stroke detection algorithms need to be assessed in terms of accuracy, computational complexity, and time demands for stroke prediction. The computational time of any method is a critical factor, as an efficient computational framework is obligatory in the acute stroke setting to administer treatment within the time-sensitive therapeutic window. Machine learning is very useful in detecting large vessel occlusion (LVO) in the diagnosis of acute stroke [151]. It is also used for the prediction of functional outcome of treatment in hemorrhagic stroke [152].

The performance of the machine learning methods depends on various parameters, e.g., image modality, image contents, and quality due to low contrast. Thus, different image enhancement techniques, such as Gaussian filter, contrast starching, and histogram equalization, are commonly adopted in medical images. The extraction of different features and in large numbers is time consuming and also makes the classification stage more complex. Hence, feature reduction methods, such as genetic algorithms, principal component analysis (PCA), and linear discriminant analysis (LDA), are commonly used. The common traditional classifiers, e.g., KNN, naive Bayes classifier, artificial neural network, SVM, and decision tree, are the most commonly used for infarct segmentation and classification. These methods achieved high-level classification accuracy. It is observed that the main limitation of studies was related to the use of small numbers of images in the training phase, which may weaken the chance of detecting infarcts. Ideally, the validation needs to be carried out on large datasets, using deep learning (DL) methods both on imaging and clinical criteria for better detection accuracy. In [124], an automated deep learning system was designed to segment the lesion in chronic stroke based on ConvNet on MRI that produced better result with Dice coefficient of 0.63. Aiming to reduce the potential for false positives, Chen et al. [125] used two CNN models and validated their results on a large clinical dataset of DWI in 741 subjects, producing Dice coefficients of 0.61 and 0.83 for the detection of small and large lesions, respectively. Choi et al. [153] used an ensemble of DNNs for the prediction of disease after an incident ischemic stroke. The method combined CNN with a logistic regression model for clinical outcome prediction. Yu et al. [126] used U-net model which achieved a dice index of 0.53 to predict ischemic stroke on DWI sequences. Thus, DL methods are now much more popular among researchers, although many challenges still exist regarding the architecture design. The DL models are very computationally expensive and require GPUs units to train the model, necessitating a huge volume of images. In practice, there is a scarcity of high-volume MRI datasets to train the deep learning models. These limitations may be overcome by establishing a common dataset to share the different modalities of MRI data of stroke across researchers from different institutions and making the dataset accessible to researchers worldwide.

## 5. Conclusions

The development of automated CAD tools is needed for the efficient detection of stroke and quantification of stroke extent, which has important therapeutic and prognostic implications. Ultimately, this will lead to timely and improved stroke management and reduced patient morbidity and mortality. Toward this aim, advancements in neuroimaging acquisition techniques, as well as the application of machine learning, play crucial roles. In this review paper, we have extensively searched for different image analysis techniques applied to detect the stroke lesion using MRI scans. In this study, we reviewed the state-of-the-art methods of segmentation and classification of brain stroke on MRI images, focusing on machine learning approaches. We concluded that integrating machine learning models and smart technology, the brain infarcts can be detected at a faster rate with higher accuracy on MRI in real-world clinical scenarios, which will be helpful in clinical decision management. In addition, the limitations of various methods and potential solutions are discussed. We believe that this work will be a valuable resource and a source of ideas and inspiration to researchers in the field.

## 6. Future Directions

To improve the performance and robustness of automated CAD tools, we propose the following recommendations. First, segmentation methods should be fully automated both to expedite the recognition of stroke infarcts as well as size. Second, a heterogeneous data structure is preferred to be adapted for model training with different modalities. Third, a large number of input images are available, and a deep learning approach can be implemented to develop fully automated systems for infarct detection and classification. Fourth, more research can focus on classifying stroke subtypes to guide specific treatment. Fifth, standardized protocols for brain MRI acquisition and image reconstruction are needed to improve the reproducibility of the observations. Sixth, an accessible cross-institutional challenge dataset comprising large number of brain MRI images should be a priority among the global research community, as it will not only facilitate metadata analysis, but will catalyze research in advanced machine learning applications. To the best of our knowledge, much less work has been reported for the automatic detection of infarcts using AI-based methods, such as deep learning techniques. AI-based methods using medical images are gradually gaining popularity and becoming a go-to technology to diagnose various diseases, e.g., diabetic retinopathy and cancer. With several researchers now working on AI-based stroke detection systems, this may be the next frontier to explore. A decision support system using AI and clinical data presenting symptoms, such as blood pressure and body mass index (BMI), has huge potential to avoid stroke occurrence or increase survival with minimal deficiency in motor functions and communicative skills. AI-based systems can support clinicians to provide insights regarding disease to accelerate diagnosis and therapy processes in the shortest possible time. This will become an essential approach to support preventive and emergency stroke care in the next five years.

## Figures and Tables

**Table 1:** Machine learning studies on detection of acute ischemic stroke lesions.

Literature	Modality	No. of Images	Segmentation Approach	Classifier	Dice Index
Prakash et al. (2008) [57]	DWI scans	13 patients; 361 images	Divergence based algorithms	ProbabilisticNN	0.6
Seghier et al. (2008) [70]	T1-weighted	10 images	Generative model	Fuzzy clustering	0.64
Subudhi et al. (2015) [78]	DWI scans	05 subjects	Hybrid FCM with thresholding	Random forest	0.79
Subudhi et al. (2018) [89]	DWI scans	142 images	Watershed and fuzzy connectedness	Random forest	0.96
Wilke et al. (2011) [115]	T1 weighted	11 subjects	Automatic lesion identification	Expert	0.49
Asit et al. (2018) [114]	DWI scan	192 images	Delaunay triangulation	Random forest	0.93
Mitra et al. (2014) [116]	Multimodal MRI	36 subjects	Bayesian-Markov random field	Random forest	0.60
Maier et al. (2015) [117]	FLAIR sequences	37 subjects	Intensity derived image features	Extra trees	0.65
Maier et al. (2015) [118]	FLAIR sequences	37 subjects	Fuzzy clustering	SVM	0.72
Griffs et al. (2016) [119]	T1 weighted	30 subjects	Probabilistic tissue segmentation	Naive Bayes classifier	0.66
Zhang et al. (2014) [120]	DWI	98 subjects	General segmentation	Random forest	0.77
Mah et al. (2014) [121]	DWI scan	435 images	Unsupervised algorithm	Neural network	0.73
Muda et al. (2015) [122]	DWI scan	30 subjects	Fuzzy C-means algorithm	Expert	0.73
Guo et al. (2015) [123]	T1 weighted	60 subjects	Unsupervised and supervised methods	SVM	0.73
Wang et al. (2016) [124]	T1 weighted	18 subjects	Deep lesion symmetry ConvNet	Neural network	0.78
Chen et al. (2017) [125]	DWI scan	741 subjects	Novel framework	CNN	0.67
Yu et al. (2020) [126]	DWI scan	182 subjects	U-net framework	Deep learning	0.53
Liu et al. (2021) [127]	DWI scan	2348 images	DAGMNet	Deep learning	0.74

## Data Availability

Not applicable.

## References

[1] GBD 2019 Stroke Collaborators (2021). Global, regional, and national burden of stroke and its risk factors, 1990–2019: A systematic analysis for the Global Burden of Disease Study 2019. Lancet Neurol..

[2] Wolfe C.D.A. (2000). The impact of stroke. Br. Med. Bull..

[3] Feigin V.L., Lawes C.M., Bennett D.A., Barker-Collo S.L., Parag V. (2009). Worldwide stroke incidence and early case fatality reported in 56 population-based studies: A systemic review. Lancet Neurol..

[4] Kamalakannan S., Gudlavalleti A.S.V., Gudlavalleti V.S.M., Goenka S., Kuper H. (2017). Incidence & prevalence of stroke in India: A systematic review. Indian J. Med. Res..

[5] Khurana S., Gourie-Devi M., Sharma S., Kushwaha S. (2021). Burden of stroke in India during 1960 to 2018: A systematic review and meta-analysis of community based surveys. Neurol. India.

[6] Tursunov D., Akbarkhodjaeva Z. (2017). Risk factors of developing transient ischemic attack. J. Neurol. Sci..

[7] Andersen K.K., Olsen T.S., Dehlendorff C., Kammersgaard L.P. (2009). Hemorrhagic and ischemic strokes compared: Stroke severity, mortality, and risk factors. Stroke.

[8] Kuriakose D., Xiao Z. (2020). Pathophysiology and treatment of stroke: Present status and future perspectives. Int. J. Mol. Sci..

[9] Bamford J., Sandercock P., Dennis M., Burn J., Warlow C. (1991). Classification and natural history of clinically identifiable subtypes of cerebral infarction. Lancet.

[10] Fisher M. (1999). Antithrombotic and thrombolytic therapy for ischemic stroke. J. Thromb. Thrombolysis.

[11] Barber P.A., Darby D.G., Desmond P.M., Gerraty R.P., Yang Q., Li T., Jolley D., Donnan G., Tress B.M., Davis S.M. (1999). Identification of major ischemic change. Diffusion-weighted imaging versus computed tomography. Stroke.

[12] Vymazal J., Rulseh A.M., Keller J., Janouskova L. (2012). Comparison of CT and MR imaging in ischemic stroke. Insights Imaging.

[13] Liang D., Bhatta S., Gerzanich V., Simard J.M. (2007). Cytotoxic edema: Mechanisms of pathological cell swelling. Neurosurg. Focus.

[14] Hagmann P., Jonasson L., Maeder P., Thiran J., Wedeen V., Meuli R. (2006). Understanding diffusion MR imaging techniques: From scalar diffusion-weighted imaging to diffusion tensor imaging and beyond. Radiographics.

[15] Moseley M., Kucharczyk J., Mintorovitch J., Cohen Y., Kurhanewicz J., Derugin N., Asgari H., Norman D. (1990). Diffusion-weighted MR imaging of acute stroke: Correlation with T2-weighted and magnetic susceptibility-enhanced MR imaging in cats. AJNR Am. J. Neuroradiol..

[16] Wardlaw J.M. (2010). Neuroimaging in acute ischemic stroke: Insights into unanswered questions of pathophysiology (review). J. Intern. Med..

[17] Han Y., Li E., Tian J., Chen J., Wang H., Dai J. (2006). The application of diffusion-and perfusion-weighted magnetic resonance imaging in the diagnosis and therapy of acute cerebral infarction. Int. J. Biomed. Imaging.

[18] Na D., Thijs V., Albers G., Moseley M., Marks M. (2004). Diffusion-weighted MR imaging in acute ischemia: Value of apparent diffusion coefficient and signal intensity thresholds in predicting tissue at risk and final infarct size. AJNR Am. J. Neuroradiol..

[19] Fiebach J.B., Schellinger P.D., Jansen O., Meyer M., Wilde P., Bender J., Schramm P., Juttler E., Oehler J., Hartmann M. (2002). CT and diffusion-weighted MR imaging in randomized order: Diffusion-weighted imaging results in higher accuracy and lower interrater variability in the diagnosis of hyperacute ischemic stroke. Stroke.

[20] Pereira R.S., Harris A.D., Sevick R.J., Frayne R. (2002). Effect of b value on contrast during diffusion-weighted magnetic resonance imaging assessment of acute ischemic stroke. J. Magn. Reson. Imaging Off. J. Int. Soc. Magn. Reson. Med..

[21] Kim H.J., Choi C.G., Lee D.H., Lee J.H., Kim S.J., Suh D.C. (2005). High-b-value diffusion-weighted MR imaging of hyperacute ischemic stroke at 1.5 t. AJNR Am. J. Neuroradiol..

[22] Delano M., Cao Y. (2002). High b-value diffusion imaging. Neuroimaging Clin..

[23] Meyer J., Gutierrez A., Mock B., Hebron D., Prager J., Gorey M., Homer D. (2000). High-b-value diffusion weighted MR imaging of suspected brain infarction. AJNR Am. J. Neuroradiol..

[24] Norouzi A., Shafry M., Rahim M., Altameem A., Saba T., Rad A.E., Rehman A., Uddin M. (2014). Medical image segmentation methods, algorithms, and applications. IETE Tech. Rev..

[25] Petrick N., Sahiner B., Armato S.G., Bert A., Correale L., Delsanto S., Freedman M.T., Fryd D., Gur D., Hadjiiski L. (2013). Evaluation of computer-aided detection and diagnosis systems. Med. Phys..

[26] Shen S., Sandham W., Granat M., Sterr A. (2005). MRI fuzzy segmentation of brain tissue using neighborhood attraction with neural network optimization. IEEE Trans. Inf. Technol. Biomed..

[27] Kabir Y., Dojat M., Scherrer B., Forbes F., Garbay C. Multimodal MRI segmentation of ischemic stroke lesions. Proceedings of the 2007 29th Annual International Conference of the IEEE Engineering in Medicine and Biology Society.

[28] Fiez J.A., Damasio H., Grabowski T.J. (2000). Lesion segmentation and manual warping to a reference brain: Intra-and interobserver reliability. Hum. Brain Ma..

[29] Takenaga T., Katsuragawa S., Uchiyama Y., Hirai T., Shiraishi J. (2012). Computerized detection of metastatic brain tumors in brain MR images. Int. J. CARS.

[30] Moher D., Liberati A., Tetzlaff J., Altman D.G. (2009). Preferred reporting items for systematic reviews and meta-analyses: The PRISMA statement. PLoS Med..

[31] Despotovic I., Goossens B., Philips W. (2015). MRI segmentation of the human brain: Challenges, methods, and applications. Comput. Math. Methods Med..

[32] Van Timmeren J., Cester D., Tanadini-Lang S., Alkadhi H., Baessler B. (2020). Radiomics in medical imaging—“How-to” guide and critical reflection. Insights Imaging.

[33] Wu Y., Wang Z., Ripplinger C.M., Sato D. (2022). Automated object detection in experimental data using Ccmbination of unsupervised and supervised methods. Front. Physiol..

[34] Sharma N., Aggarwal L.M. (2010). Automated medical image segmentation techniques. J. Med. Phys..

[35] Wang Y., Xiang S., Pan C., Wang L., Meng G. (2013). Level set evolution with locally linear classification for image segmentation. Pattern Recogn..

[36] Alpert S., Galun M., Brandt A., Basri R. (2012). Image segmentation by probabilistic bottom-up aggregation and cue integration. IEEE Trans. Pattern Anal. Mach. Intell..

[37] Saad N.M., Bakar S.A., Muda S., Mokji M.M. Brain lesion segmentation of diffusion-weighted MRI using thresholding technique. Proceedings of the 5th Kuala Lumpur International Conference on Biomedical Engineering 2011.

[38] Caldairou B., Passat N., Habas P.A., Studholme C., Rousseau F. (2011). A non-local fuzzy segmentation method: Application to brain MRI. Pattern Recogn..

[39] Boykov Y., Veksler O., Zabih R. (2001). Fast approximate energy minimization via graph cuts. IEEE Trans. Pattern Anal. Mach. Intell..

[40] McGrath H., Li P., Dorent R., Bradford R., Saeed S., Bisdas S., Ourselin S., Shapey J., Vercauteren T. (2020). Manual segmentation versus semi-automated segmentation for quantifying vestibular schwannoma volume on MRI. Int. J. CARS.

[41] Dastidar P., Heinonen T., Ahonen J.P., Jehkonen M., Molnar G. (2000). Volumetric measurements of right cerebral hemisphere infarction: Use of a semiautomatic MRI segmentation technique. Comput. Biol. Med..

[42] Matesin M., Loncaric S., Petravic D. A rule-based approach to stroke lesion analysis from CT brain images. Proceedings of the 2nd International Symposium on Image and Signal Processing and Analysis. In conjunction with 23rd International Conference on Information Technology Interfaces.

[43] Usinskas E., Pranckeviciene A., Wittenberg T., Hastreiter P., Tomand B. (2002). Automatic ischemic stroke segmentation using various techniques. Neural Netw. Soft Comput..

[44] Meilunas M., Usinskas A., Kirvaitis R., Dobrovolskis R. (2003). Automatic contouring of segmented human brain ischemic stroke region on CT images. Math. Model. Anal..

[45] Muir K.W., Baird-Gunning J., Walker L., Baird T., McCormick M., Coutts S.B. (2007). Can the ischemic penumbra be identified on noncontrast CT of acute stroke?. Stroke.

[46] James J.R., Yoder K.K., Osuntokun O., Kalnin A., Bruno A., Morris E.D. (2006). A supervised method for calculating perfusion/diffusion mismatch volume in acute ischemic stroke. Comput. Biol. Med..

[47] Mangla A.R., Kolar B., Almast J., Ekholm S.E. (2011). Border zone infarcts: Pathophysiologic and imaging characteristics. Radiographics.

[48] Sezgin M., Sankur B. (2004). Survey over image thresholding techniques and quantitative performance evaluation. J. Electron. Imaging.

[49] Martel A., Allder S., Delay G., Morgan P., Moody A. Measurement of infarct volume in stroke patients using adaptive segmentation of diffusion weighted MR images. Proceedings of the International Conference on Medical Image Computing and Computer-Assisted Intervention.

[50] Usinskas A., Dobrovolskis R.A., Tomandl B. (2004). Ischemic stroke segmentation on CT images using joint features. Informatica.

[51] Paing M.P., Tungjitkusolmun S., Bui T.H., Visitsattapongse S., Pintavirooj C. (2021). Automated Segmentation of Infarct Lesions in T1-Weighted MRI Scans Using Variational Mode Decomposition and Deep Learning. Sensors.

[52] Liew A.C., Yan H. (2003). An adaptive spatial fuzzy clustering algorithm for 3-D MR image segmentation. IEEE Trans. Med. Imaging.

[53] Van Leemput K., Maes F., Vandermeulen D., Suetens P. (1999). Automated model-based tissue classification of MR images of the brain. IEEE Trans. Med. Imaging.

[54] Pham D.L., Prince J.L. (1999). Adaptive fuzzy segmentation of magnetic resonance images. IEEE Trans. Med. Imaging.

[55] Maldjian J.A., Chalela J., Kasner S.E., Liebeskind D., Detre J.A. (2001). Automated CT segmentation and analysis for acute middle cerebral artery stroke. AJNR Am. J. Neuroradiol.

[56] Li W., Tian J., Li E., Dai J. (2004). Robust unsupervised segmentation of infarct lesion from diffusion tensor MR images using multiscale statistical classification and partial volume voxel reclassification. NeuroImage.

[57] Prakash K.N., Gupta V., Jianbo H., Nowinski W.L. (2006). Identification, segmentation, and image property study of acute infarcts in diffusion-weighted images by using a probabilistic neural network and adaptive gaussian mixture model. Int. J. Comput. Assist. Radiol. Surg..

[58] Hevia-Montiel N., Jimenez-Alaniz J.R., Medina-Banuelos V., Yanez-Suarez O., Rosso C., Samson Y., Baillet S. Robust nonparametric segmentation of infarct lesion from diffusion-weighted MR images. Proceedings of the 2007 29th Annual International Conference of the IEEE Engineering in Medicine and Biology Society.

[59] Gupta V., Prakash B., Nowinski W.L. (2008). Automatic and rapid identification of infarct slices and hemisphere in DWI scans. Acad. Radiol..

[60] Shen S., Szameitat A.J., Sterr A. (2008). Detection of infarct lesions from single MRI modality using inconsistency between voxel intensity and spatial location-a 3-D automatic approach. IEEE Trans. Inf. Technol. Biomed..

[61] Wang J., Kong J., Lu Y., Qi M., Zhang B. (2008). A modified FCM algorithm for MRI brain image segmentation using both local and non-local spatial constraints. Comput. Med. Imaging Graph..

[62] Ramathilagama S., Pandiyarajanc R., Sathyac A., Devic R., Kannan S.R. (2011). Modified fuzzy-means algorithm for segmentation of T1-T2-weighted brain MRI. J. Comput. Appl. Math..

[63] Sulaiman S.N., Isa N.A.M. (2010). Adaptive fuzzy-K-means clustering algorithm for image segmentation. IEEE Trans. Consum. Electron..

[64] Yang Y. (2007). Image segmentation by fuzzy c-means clustering algorithm with a novel penalty term. Comput. Inform..

[65] Hui C., Zhou Y.X., Narayana P. (2010). A fast algorithm for calculation of inhomogeneity gradient in MRI data. J. Magn. Reson. Imaging.

[66] Yogita K., Dubey K., Mushrif M. (2016). FCM Clustering Algorithms for Segmentation of Brain MR Images. Adv. Fuzzy Syst..

[67] Yang Z., Chung F.-L., Shitong W. (2009). Robust fuzzy clustering-based image segmentation. Appl. Soft Comput..

[68] Xue J.H., Pizurica A., Philips W., Kerre E., van de Walle R., Lemahieu I. (2003). An integrated method of adaptive enhancement for unsupervised segmentation of MRI brain images. Pattern Recog. Lett..

[69] Jayachitra S., Prasanth A. (2021). Multi-Feature Analysis for Automated Brain Stroke Classification Using Weighted Gaussian Naïve Bayes Classifier. J. Circuits Syst. Comput..

[70] Seghier M.L., Ramlackhansingh A., Crinion J., Leff A.P., Price C.J. (2008). Lesion identification using unified segmentation-normalization models and fuzzy clustering. Neuroimage.

[71] Zheng Y., Jeon B., Xu D., Wu Q.M., Zhang H. (2015). Image segmentation by generalized hierarchical fuzzy c-means algorithm. J. Intell. Fuzzy Syst..

[72] Shah B., Shah S., Kosta Y.P. (2012). Novel improved fuzzy c-mean algorithm for MR-Image segmentation. Int. J. Soft Comput. Eng..

[73] He L., Wen Y., Wan M., Liu S. (2014). Multi-channel features based automated segmentation of diffusion tensor imaging using an improved FCM with spatial constraints. Neurocomputing.

[74] Ahmed M., Yamany S., Mohamed N., Farag A., Moriarty T. (2002). A modified fuzzy c-means algorithm for bias field estimation and segmentation of MRI data. IEEE Trans. Med. Imag..

[75] Liew A.W.C., Yan H., Law N.F. (2005). Image segmentation based on adaptive cluster prototype estimation. IEEE Trans. Fuzzy Syst..

[76] Krinidis S., Chatzis V. (2010). A robust fuzzy local information c-means clustering algorithm. IEEE Trans. Image Process..

[77] Assia C., Yazid C., Said M. (2015). Segmentation of brain MRIs by support vector machine: Detection and characterization of strokes. J. Mech. Med. Biol..

[78] Subudhi A., Jena S.S., Sabut S. (2019). Automated detection of brain stroke in MRI with hybrid fuzzy c-means clustering and random forest classifier and applications. Int. J. Comput. Intell. Appl..

[79] Beucher S., Meyer F., Dougherty E. (1993). The morphological approach to segmentation: The watershed transform. Mathematical Morphology in Image Processing.

[80] Moga A.N., Gabbouj M. (1997). Parallel image component labelling with watershed transformation. IEEE Trans. Pattern Anal. Mach. Intell..

[81] Hagyard D., Razaz M., Atkin P. Analysis of watershed algorithms for grayscale images. Proceedings of the 3rd IEEE International Conference on Image Processing.

[82] Karantzalos K., Argialas D. (2006). Improving edge detection and watershed segmentation with anisotropic. Int. J. Remote Sens..

[83] Benson C.C., Lajish V.L., Kumar R. Brain tumor extraction from MRI brain images using marker based watershed algorithm. Proceedings of the 2015 International Conference on Advances in Computing, Communications and Informatics (ICACCI).

[84] Warscotte V., Macq B., Thiran J., Michel C. Accurate segmentation of 3-d magnetic resonance images of the head using a directional watershed transform. Proceedings of the 17th International Conference of the Engineering in Medicine and Biology Society.

[85] Grau V., Mewes A.J., Alcaniz M., Kikinis R., Warfield S.K. (2004). Improved watershed transform for medical image segmentation using prior information. IEEE Trans. Med. Imaging.

[86] Liang Y., Fu J. (2019). Watershed algorithm for medical image segmentation based on morphology and total variation model. Int. J. Pattern Recognit. Artif. Intell..

[87] Macenko M., Celenk M., Ma L. Lesion detection using morphological watershed segmentation and model based inverse filtering. Proceedings of the 18th International Conference on Pattern Recognition (ICPR’06).

[88] Letteboer M.J., Olsen O.F., Dam E.B., Willems P.A., Viergever M.A., Niessen W.J. (2004). Segmentation of tumors in magnetic resonance brain images using an interactive multiscale watershed algorithm. Acad. Radiol..

[89] Subudhi A., Jena S., Sabut S. (2018). Delineation of the ischemic stroke lesion based on watershed and relative fuzzy connectedness in brain MRI. Med Biol Eng Comput..

[90] Lotjonen J.M., WolzKoikkalainen R., Thurfjell L., Waldemar G., Soininen H., Rueckert D. (2010). Fast and robust multi-atlas segmentation of brain magnetic resonance images, Alzheimer’s disease neuroimaging initiative. Neuroimage.

[91] Si T., De A., Bhattacharjee A.K. (2015). Grammatical swarm based segmentation methodology for lesion segmentation in brain MRI. Int. J. Comput. Appl..

[92] De A., Das R.L., Bhattacharjee A.K., Sharma D. Masking based segmentation of diseased MRI images. Proceedings of the 2010 International Conference on Information Science and Applications.

[93] Nabizadeh N., John N., Wright C. (2014). Histogram-based gravitational optimization algorithm on single MR modality for automatic brain lesion detection and segmentation. Expert. Syst. Appl..

[94] Karthik R., Menaka R. (2017). A multi-scale approach for detection of ischemic stroke from brain MR images using discrete curvelet transformation. Measurement.

[95] Pham T.X., Siarry P., Oulhadj H. (2018). Integrating fuzzy entropy clustering with an improved PSO for MRI brain image segmentation. Appl. Soft Comput..

[96] Ghosh N., Recker R., Shah A., Bhanu B., Obenaus S.A. (2011). Automated ischemic lesion detection in a neonatal model of hypoxic ischemic injury. J. Magn. Reson. Imaging.

[97] Kulkarni R.V., Venayagamoorthy G.K. (2010). Bio-inspired algorithms for autonomous deployment and localization of sensor. IEEE Trans. Syst..

[98] Kennedy J., Eberhart R. Particle Swarm Optimization. Proceedings of the ICNN’95-International Conference on Neural Networks.

[99] Subudhi A., Sahoo S., Biswal P., Sabut S.K. (2018). Segmentation and classification of ischemic stroke using optimized features in brain MRI. Biomed. Eng. Appl. Basis Commun..

[100] Couceiro M.S., Ferreira N.F., Machado J.T. (2016). Fractional Order Darwinian Particle Swarm Optimization.

[101] Lorenzo-Valdes M., Sanchez-Ortiz G.I., Elkington A.G., Mohiaddin R.H., Rueckert D. (2004). Segmentation of 4D cardiac MR images using a probabilistic atlas and the EM algorithm. Med. Image Anal..

[102] Meng X.L., Rubin D.B., Donal (1993). Maximum likelihood estimation via the ECM algorithm: A general framework. Biometrika.

[103] Yoon U.C., Kim J.S., Kim I.Y., Kim S.I. (2001). Adaptable fuzzy C-Means for improved classification as a preprocessing procedure of brain parcellation. J. Digit. Imaging.

[104] Niu M., Zhao Q., Li H., Huang D.S., Jo K.H., Wang L. (2014). Comparison of EM-based algorithms and image segmentation evaluation. International Conference on Intelligent Computing.

[105] Huang Z.K., Liu D.H. Segmentation of color image using EM algorithm in HSV color space. Proceedings of the 2007 International Conference on Information Acquisition.

[106] Mahjoub M.A., Kalti K. (2011). Image segmentation by adaptive distance based on EM algorithm. Int. J. Adv. Comput. Sci. Appl..

[107] Marroquin J.L., Vemuri B.C., Botello S., Calderon E., Fernandez-Bouzas A. (2002). An accurate and efficient Bayesian method for automatic segmentation of brain MRI. IEEE Trans. Med. Imaging.

[108] Tian G., Xia Y., Zhang Y., Feng D. (2011). Hybrid genetic and variational expectation-maximization algorithm for Gaussian-mixture-model-based brain MR image segmentation. IEEE Trans. Inf. Technol. Biomed..

[109] Prakash M., Kumari R.S. (2017). Spatial Fuzzy C means and expectation maximization algorithms with bias correction for segmentation of MR brain images. J. Med Syst..

[110] Kwon G.R., Basukala D., Lee S.W., Lee K.H., Kang M. (2016). Brain image segmentation using a combination of expectation-maximization algorithm and watershed transform. Int. J. Imaging Syst. Technol..

[111] Rouainia M., Medjram M.S., Doghmane N. (2008). Brain MRI Segmentation and lesions detection by EM algorithm. Int. J. Med. Health Sci..

[112] Pennisi A., Bloisi D.D., Nardi D., Giampetruzzi A.R., Mondino C., Facchiano A. (2016). Skin lesion image segmentation using Delaunay Triangulation for melanoma detection. Comput. Med. Imaging Graph..

[113] Häfner M., Liedlgruber M., Uhl A., Vécsei A., Wrba F. (2012). Delaunay triangulation-based pit density estimation for the classification of polyps in high-magnification chromo-colonoscopy. Comput. Methods Prog. Biomed..

[114] Subudhi A., Acharya U.R., Dash M., Jena S., Sabut S. (2018). Automated approach for detection of ischemic stroke using Delaunay Triangulation in brain MRI images. Comput. Biol. Med..

[115] Wilke M., de Haan B., Juenger H., Karnath H.O. (2011). Manual, semi-automated, and automated delineation of chronic brain lesions: A comparison of methods. Neuroimage.

[116] Mitra J., Bourgeat P., Fripp J., Ghose S., Rose S., Salvado O., Connelly A., Campbell B., Palmer S., Sharma G. (2014). Lesion segmentation from multimodal MRI using random forest following ischemic stroke. Neuroimage.

[117] Maier O., Wilms M., von der Gablentz J., Kramer U.M., Munte T.F., Handels H. (2015). Extra Tree forests for sub-acute ischemic stroke lesion segmentation in MR sequences. J. Neurosci. Methods.

[118] Maier O., Wilms M., Gablentz J., Kramer U., Handels H. (2014). Ischemic stroke lesion segmentation in multi-spectral MR images with support vector machine classifiers. Medical Imaging, Computer-Aided Diagnosis.

[119] Griffis J.C., Allendorfer J.B., Szaflarski J.P. (2016). Voxel-based Gaussian naive Bayes classification of ischemic stroke lesions in individual T1-weighted MRI scans. J. Neurosci. Methods.

[120] Zhang X., Elazab A., Qingmao H. Segmentation of hyper-acute cerebral infarct based on random forest and sparse coding from diffusion weighted imaging. Proceedings of the 2017 39th Annual International Conference of the IEEE Engineering in Medicine and Biology Society (EMBC).

[121] Mah Y.H., Jager R., Kennard R., Husain M., Nachev P. (2014). A new method for automated high-dimensional lesion segmentation evaluated in vascular injury and applied to the human occipital lobe. Cortex.

[122] Muda A.F., Saad N.M., Abu-Bakar S.R., Muda A.S., Abdullah A.R. (2015). Brain lesion segmentation using fuzzy C-means on diffusion-weighted imaging. ARPN J. Eng. Appl. Sci..

[123] Guo D., Fridriksson J., Fillmore P., Rorden C., Yu H., Zheng K., Wang S. (2015). Automated lesion detection on MRI scans using combined unsupervised and supervised methods. BMC Med. Imaging.

[124] Wang Y., Katsaggelos A.K., Wang X., Parrish T.B. A deep symmetry convnet for stroke lesion segmentation. Proceedings of the 2016 IEEE International Conference on Image Processing (ICIP).

[125] Chen L., Bentley P., Rueckert D. (2017). Fully automatic acute ischemic lesion segmentation in DWI using convolutional neural networks. Neuroimage Clin..

[126] Yu Y., Xie Y., Thamm T., Gong E., Ouyang J., Huang C., Christensen S., Marks M.P., Lansberg M.G., Albers G.W. (2020). Use of Deep Learning to Predict Final Ischemic Stroke Lesions From Initial Magnetic Resonance Imaging. JAMA Netw. Open.

[127] Liu C.F., Hsu J., Xu X., Ramachandran S., Wang V., Miller M.I., Hillis A.E., Faria A.V., The STIR and VISTA Imaging investigators (2021). Deep learning-based detection and segmentation of diffusion abnormalities in acute ischemic stroke. Commun. Med..

[128] Abedi V., Goyal N., Tsivgoulis G., Hosseinichimeh N., Hontecillas R., Bassaganya-Riera J., Elijovich L., Metter J.E., Alexandrov A.W., Liebeskind D.S. (2017). Novel screening tool for stroke using artificial neural network. Stroke.

[129] Huang S., Shen Q., Duong T.Q. (2010). Artificial neural network prediction of ischemic tissue fate in acute stroke imaging. J. Cereb. Blood Flow Metab..

[130] Ledezma C.J., Wintermark M. (2009). Multimodal CT in stroke imaging: New concepts. Radiol. Clin. N. Am..

[131] Kasasbeh A.S., Christensen S., Parsons M.W., Campbell B., Albers G.W., Lansberg M.G. (2019). Artificial neural network computer tomography perfusion prediction of ischemic core. Stroke.

[132] Chyzhyk D., Dacosta-Aguayo R., Mataro M., Grana M. (2015). An active learning approach for stroke lesion segmentation on multimodal MRI data. Neurocomputing.

[133] Ho K.C., Speier W., Zhang H., Scalzo F., El-Saden F., Arnold C.W. (2019). A Machine Learning Approach for Classifying Ischemic Stroke Onset Time From Imaging. IEEE Trans. Med. Imaging.

[134] Amini L., Azarpazhouh R., Farzadfar M., Mousavi S.A., Jazaieri F., Khorvash F., Norouzi R., Toghianfar N. (2013). Prediction and control of stroke by data mining. Int. J. Prev. Med..

[135] Tomita N., Jiang S., Maeder M.E., Hassanpour S. (2020). Automatic post-stroke lesion segmentation on MR images using 3D residual convolu-tional neural network. Neuroimage Clin..

[136] Bhattacharya S., Maddikunta P.K.R., Hakak S., Khan W.Z., Bashir A.K., Jolfaei A., Tariq U. (2020). Antlion re-sampling based deep neural network model for classification of imbalanced multimodal stroke dataset. Multimed. Tools Appl..

[137] Maier O., Schroder C., Forkert N.D., Martinetz T., Handels H. (2016). Correction: Classifiers for ischemic stroke lesion segmentation: A comparison study. PLoS ONE.

[138] Guibas L.J., Stolfi J. (1985). Primitives for the manipulation of general subdivisions and the computation of Voronoi diagrams. ACM Trans. Graphics.

[139] Spanel M., Krsek P., Svub M., Stancl V., Siler O., Kropatsch W.G., Kampel M., Hanbury A. (2007). Delaunay-Based Vector Segmentation of Volumetric Medical Images. Computer Analysis of Images and Patterns.

[140] Akkus Z., Galimzianova A., Hoogi A., Rubin D.L., Erickson B.J. (2017). Deep Learning for Brain MRI Segmentation: State of the Art and Future Directions. J. Digit. Imaging.

[141] Nayak D.R., Das D., Dash R., Majhi S., Majhi B. (2020). Deep extreme learning machine with leaky rectified linear unit for multiclass classification of pathological brain images. Multimed. Tools Appl..

[142] Leea E.J., Kima Y.H., Kimb N., Kanga D.W. (2017). Deep into the brain: Artificial intelligence in stroke imaging. J. Stroke.

[143] Soun J.E., Chow D.S., Nagamine M., Takhtawala R.S., Filippi C.G., Yu W., Chang P.D. (2021). Artificial Intelligence and acute stroke imaging. AJNR Am. J. Neuroradiol..

[144] Feng R., Badgeley M., Mocco J., Oermann E.K. (2018). Deep learning guided stroke management: A review of clinical applications. J. Neurointerv Surg..

[145] Haskins G., Kruger U., Yan P. (2020). Deep learning in medical image registration: A survey. Mach. Vis. Appl..

[146] Kaur T., Gandhi T.K. (2020). Deep convolutional neural networks with transfer learning for automated brain image classification. Mach. Vis. Appl..

[147] Winzeck S., Mocking S.J.T., Bezerra R., Bouts M.J.R.J., McIntosh E.C., Diwan I., Garg P., Chutinet A., Kimberly W.T., Copen W.A. (2019). Ensemble of convolutional neural networks improves automated segmentation of acute ischemic lesions using multiparametric diffusion-weighted MRI. AJNR Am. J. Neuroradiol..

[148] Xue Y., Farhat F.G., Boukrina O., Barrett A.M., Binder J.R., Roshan U.W., Graves W.W. (2020). A multi-path 2.5 dimensional convolutional neural network system for segmenting stroke lesions in brain MRI images. Neuroimage Clin..

[149] Bridge C.P., Bizzo B.C., Hillis J.M., Chin J.K., Comeau D.S., Gauriau R., Macruz F., Pawar J., Noro F.T.C., Sharaf E. (2022). Development and clinical application of a deep learning model to identify acute infarct on magnetic resonance imaging. Sci. Rep..

[150] Chang P.D., Kuoy E., Grinband J., Weinberg B.D., Thompson M., Homo R., Chen J., Abcede H., Shafie M., Sugrue L. (2018). Hybrid 3D/2D convolutional neural network for hemorrhage evaluation on head CT. AJNR Am. J. Neuroradiol..

[151] Murray N.M., Unberath M., Hager G.D., Hui F.K. (2020). Artificial intelligence to diagnose ischemic stroke and identify large vessel occlusions: A systematic review. J. Neurointerv. Surg..

[152] Guo R., Zhang R., Liu R., Liu Y., Li H., Ma L., He M., You C., Tian R. (2022). Machine learning-based approaches for prediction of patients’ functional outcome and mortality after spontaneous intracerebral hemorrhage. J. Pers. Med..

[153] Choi Y., Kwon Y., Lee H., Kim B.J., Paik M.C., Won J.H. (2016). Ensemble of deep convolutional neural networks for prognosis of ischemic stroke. International Workshop on Brainlesion: Glioma, Multiple Sclerosis, Stroke and Traumatic Brain Injuries.

